# Integration of clinical information for identification of gangrenous or perforated appendicitis in children: a multicenter retrospective cohort study with external validation

**DOI:** 10.1186/s12887-026-07314-3

**Published:** 2026-07-21

**Authors:** Honglong Ma, Junjun Ouyang, Jie Liu, Rui Tang, Xiao Wang, Zhenjie Chu, Zhiyuan Jin, Bangzhi Sui, Guangyu Wang, Jin Chen, Lang Cheng

**Affiliations:** 1 Department of Pediatric Surgery, The First Affiliated Hospital of Wannan Medical College, No. 2, Zheshan Road, Jinghu District, Wuhu, 241001 People’s Republic of China; 2 Department of Pediatric Surgery, Anqing Municipal Hospital, No. 87, Tianzhu Road, Yixiu District, Anqing, 246003 People’s Republic of China; 3 Department of Pediatric Orthopaedics, The First Affiliated Hospital of Wannan Medical College, No. 2, Zheshan Road, Jinghu District, Wuhu, 241001 People’s Republic of China; 4 Department of Urology, The First Affiliated Hospital of Wannan Medical College, No. 2, Zheshan Road, Jinghu District, Wuhu, 241001 People’s Republic of China

**Keywords:** Appendicitis, Gangrene, Perforation, Complicated, Pediatrics, Nomogram

## Abstract

**Background:**

Acute complicated appendicitis in children is associated with substantial morbidity, including prolonged hospitalization, abscess formation, higher rates of surgical site infection, postoperative ileus, chronic abdominal pain, and increased need for reoperation. Preoperative identification of pediatric patients at elevated risk for complicated appendicitis is essential to optimize surgical management and improve patient and family counseling. This study aimed to identify reliable preoperative predictors of acute complicated appendicitis in childrenand to develop an accurate, clinically practical predictive nomogram.

**Methods:**

We conducted a retrospective analysis of 735 pediatric patients diagnosed with acute appendicitis who underwent surgical treatment at our institutionbetween October 2019 and September 2025. Patients were randomly divided into a training cohort and an internal validation cohort in a 7:3 ratio. An external validation cohort included 450 patients from another institution. Feature selection was performed using LASSO (Least Absolute Shrinkage and Selection Operator) regression to identify the most significant predictive variables. A multivariate logistic regression model was then developed, incorporating the coefficientsof these selected features to create a predictive nomogram. The model’s performance was rigorously assessed across all cohorts in terms of discriminative ability, calibration, and clinical utility.

**Results:**

Nine significant preoperative predictors were identified: neutrophil-to-lymphocyte ratio, C-reactive protein level, serum chloride, fibrinogen, appendicolith, free peritoneal effusion, onset time, peritoneal irritation signs, and fever(≥ 38 °C). The nomogram demonstrated robust discriminatory performance, with area under the curve (AUC) values of 0.922, 0.916, and 0.906 in the training, internal validation, and external validation cohorts, respectively. Additionally, the model showed excellent calibration and significant clinical utility, as demonstrated by decision curve analysis across all cohorts.

**Conclusions:**

This study successfully developed and validated a high-performance nomogram that effectively distinguishes between acute complicated and uncomplicated appendicitis in children. This tool may assist clinicians in early risk stratification and could contribute to improved patient outcomes.

**Supplementary Information:**

The online version contains supplementary material available at https://doi.org/10.1186/s12887-026-07314-3.

## Introduction

The global incidence of acute appendicitis is rising, imposing substantial clinical and economic burdens on healthcare systems worldwide [1]. The condition is notably more common among children, with approximately 30% to 40% of pediatric cases progressing to acute complicated appendicitis (ACA) [2]. While some studies suggest that conservative management can be a viable alternative to surgery for certain cases of acute uncomplicated appendicitis (AUA) [3–5], ACA nearly always requires urgent surgical intervention [6]. Delayed intervention in ACA patients increases the risk of morbidity, including abscess formation, perforation, ileus, intra-abdominal infection, and sepsis [7, 8]. Such delays are also associated with prolonged hospitalization and higher healthcare costs [9], alongside potential long-term effects on children’s growth, development, and quality of life [10, 11].Therefore, accurately distinguishing ACA from AUA in children is critically important in clinical practice.

However, existing tools have notable limitations. In recent years, clinical scoring systems such as the Pediatric Appendicitis Score (PAS), the modified Heidelberg Appendicitis Score (HAS), and the Alvarado score have been developed and are effective for diagnosing appendicitis, yet they show limited accuracy in predicting its severity, particularly in children [12, 13]. Concurrently, several studies have proposed scoring models to predict ACA in pediatric patients. However, due to limitations such as a restricted set of variables, small sample sizes, or reliance on data from a single medical center, these models have not undergone extensive external validation [14, 15].

Therefore, we developed a predictive model for pediatric ACA by integrating LASSO regression and binary logistic regression, using data from a national tertiary medical center in central and eastern China. The aim was to identify highly relevant predictive variables. After assessing the model’s credibility, stability, and generalizability, we presented it visually as a nomogram. We further validated the model using an independent cohort from a tertiary referral medical center. Ultimately, this nomogram-based prediction tool enables clinical staff to rapidly screen for ACA in children, facilitating early identification, timely intervention, and improved prognosis.

## Materials and methods

### Study design and population

This study enrolled 735 consecutive pediatric patients with confirmed acute appendicitis who were admitted to The First Affiliated Hospital of Wannan Medical College for surgical intervention between October 2019 and September 2025. The study was conducted in accordance with the Declaration of Helsinki and was approved by the Ethics Committees of The First Affiliated Hospital of Wannan Medical College and Anqing Municipal Hospital. The dataset was randomly divided into training and internal validation cohorts in a 7:3 ratio using a random seed. An external validation cohort was also established, consisting of allconsecutive pediatric patients with acute appendicitis admitted for surgery at Anqing Municipal Hospital—another tertiary referral center—between September 2021 and September 2024. Due to the retrospective design and the use of anonymized data, the requirement for written informed consent was waived by both ethic committees. Patients with surgical indications underwent laparoscopic appendectomy at both centers [16]. Patients managed non-operatively were excluded from this study because pathological confirmation, which is required to definitively classify appendicitis severity (ACA vs. AUA), was unavailable—a recognized limitation of the retrospective design. Both institutions are tertiary referralcenters located in the same province, sharing standardized clinical pathways forpediatric acute abdomen and similar indications for laparoscopic appendectomy.The imaging protocols at the external center also strictly adhered to low-dose pediatric CT guidelines. Although there is a temporal overlap between the derivation and external validation cohorts, this concurrent design effectively demonstrates the spatial transportability of the model across different healthcare facilities operating under contemporary clinical practices.

Inclusion criteria were: (1) pediatric patients with a diagnosis of appendicitis confirmed by pathological examination following laparoscopic appendectomy; (2) age ≤ 14 years. This age cutoff was selected to establish a more homogeneous cohort of pre-pubertal and early-adolescent patients, consistent with the population typically managed by the pediatric surgery services at the participating institutions; (3) availability of complete clinical records.

Exclusion criteria were: (1) concurrent inflammatory conditions (e.g., respiratoryor urinary tract infections) or chronic wasting diseases, including malignancies; (2) known immunodeficiency disorders; (3) diagnosis of chronic appendicitis, acute exacerbation of chronic appendicitis, or periappendiceal abscess, as no surgery was performed in these cases; (4) intraoperative findings of other conditions, such as Meckel’s diverticulum, omental infarction, or ovarian cysts; (5) history of major trauma or prior major surgery.

Patients were divided into the ACA group (*n* = 363) and the AUA group (*n* = 372.Clinical, laboratory, and imaging data were compared between the two groups to identify any differences. All patient data were independently collected and integrated by two senior attending physicians. For the purpose of this study, imaging evaluations for all patients were independently conducted by two senior radiologists in a double-blind manner strictly. The classification of appendicitis severity was strictly based on a combination of intraoperative findings and final histopathological reports. The ACA was defined by the presence of at least one of the following criteria: (1) intraoperative visualization of appendiceal gangrene, macroscopic perforation, or purulent/fecal peritonitis, confirmed by consensus between two experienced pediatric surgeons; or (2) histopathological evidence of transmural necrosis or perforation extending through the muscularis propria, confirmed by two senior pathologists, Similarly. Any discrepancies between the two reviewers were resolved through discussion to reach a consensus. Conversely, the AUA was defined as acute simple or suppurative appendicitis, characterized by an inflamed, intact appendix without any macroscopic or microscopic evidence of gangrene, perforation, or periappendiceal purulent fluid. Patients with periappendiceal abscesses were excluded due to their typical non-surgical management. Clinical data were retrospectively collected using a standardized case report form, encompassing demographic characteristics, medical history, physical examination findings, laboratory results, and imaging studies.

Following consent from each patient’s legal guardian, all patients underwent abdominal computed tomography (CT) examinations (Philips 256-slice Spectral CT; GE 64-slice CT) at two different hospitals. The indications for CT at the two institutions during the study period included: (1) equivocal clinical presentations with inconclusive initial ultrasound findings (often performed at referring primary care centers); (2) severe obesity or excessive bowel gas limiting ultrasound evaluation; and (3) high clinical suspicion of perforation or abscess requiring precise anatomical mapping prior to surgery. These examinations were conducted primarily to distinguish between ACA and AUA cases, in accordance with institutional clinical practice during the study period [17]. Scanning parameterswere individually adjusted, with tube voltage ranging from 80 to 100 KV and automatic tube current modulation based on patient size. This protocol was specifically designed and implemented to minimize radiation exposure while maintaining diagnostic image quality. The effective radiation dose was consistently maintained below 8 millisieverts (mSv) for all patients, and for a substantial proportion of the cohort, the dose was further reduced to less than 6 mSv. All imaging data were retrospectively collected from the Picture Archiving and Communication System (PACS) for analysis.

### Data collection

We collected potential predictive factors from all patients prior to surgery. These included gender, age, body mass index (BMI), onset time, fever(≥ 38 °C), emesis, peritoneal irritation signs, leukocyte count, neutrophil count, lymphocyte count, neutrophil-lymphocyte ratio (NLR), monocyte count, hemoglobin, platelet count, C-reactive protein (CRP), serum potassium, serum sodium, serum chloride, fibrinogen, lactic dehydrogenase, lipase, carbon dioxide combining power, bloodtypes, maximal appendix diameter, appendicolith, free peritoneal effusion, periappendiceal fat blurring, local abscess, ileus, and enlarged lymph nodes in the ileocecal region. This comprehensive set of variables was selected based on a review of the existing literature and clinical experience, with the aim of exploringthe value of various markers in reflecting the systemic inflammatory response, metabolic disturbances, and organ stress associated with severe infection in ACA [18, 19]. Outcome data (dependent variables) were also collected.

### Statistical analysis

Baseline characteristics were compared between the groups. Continuous variables with a normal distribution are presented as mean ± standard deviation, while non-normally distributed data are reported as median (interquartile range). The 95% confidence intervals (CIs) were calculated using the profile likelihood method. Missing lactate dehydrogenase values were present in 31 cases; these were imputed using the random forest method, a widely used approach for handling missing data [20]. For univariate analyses, categorical variables were analyzed using the chi-square test or Fisher’s exact test, as appropriate. Continuous variables were assessed with Student’s t-test or the Mann–Whitney U test, depending on their distribution. In the training cohort, LASSO logistic regression was used for multivariable analysis to identify independent predictors and construct apredictive model, as it effectively handles a relatively large number of potentially correlated predictors. Prior to LASSO analysis, all continuous variables werestandardized (converted to Z-scores) to eliminate the influence of different measurement scales. The LASSO method applies regularization to constrain partial regression coefficients, thereby mitigating multicollinearity and reducing the riskof overfitting in high-dimensional datasets. Consequently, it produces more stable and parsimonious results compared to traditional stepwise regression. Its output, a simple linear model, is also more transparent and clinically interpretable than “black-box” machine learning methods such as random forests. The optimalpenalty parameter (lambda, λ) was selected through 10-fold cross-validation based on the value yielding the minimum deviance within one standard error. Variables with non-zero coefficients were retained in the final model.

Subsequently, to ensure the model’s robustness and generalizability, a three-stage validation process was conducted. During model development in the training cohort, a 10-fold cross-validation was embedded within the LASSO regression to determine the optimal penalty parameter (λ) and mitigate overfitting. Second, internal validation was conducted using the 30% hold-out cohort (*n* = 220) from the primary institution. Third, external validation was performed on a completely independent cohort (*n* = 450) from a separate tertiary center. Across all cohorts, model performance was systematically evaluated across the three dimensions: (1) Discrimination, quantified by the area under the receiver operating characteristic (ROC) curve (AUC) to assess discriminatory power, where 0.5 indicates no discriminative ability and 1.0 indicates perfect discrimination, using ROC curve analysis with the proc package in R software; (2) Calibration, assessed visually via calibration curves and statistically using the Hosmer–Lemeshow test with the rms package; and (3) Clinical utility, evaluated through decision curve analysis (DCA) to estimate the net benefit at various threshold probabilities withthe dcurves package. To facilitate clinical application, an interactive web-based dynamic nomogram was created using the shiny package. A two-tailed *p*-value < 0.05 was considered statistically significant. All statistical analyses were performed using R software (version 4.2.2) and MSTATA software (www.mstata.com).

## Results

### Baseline characteristics of the study cohort

**Fig. 1:**
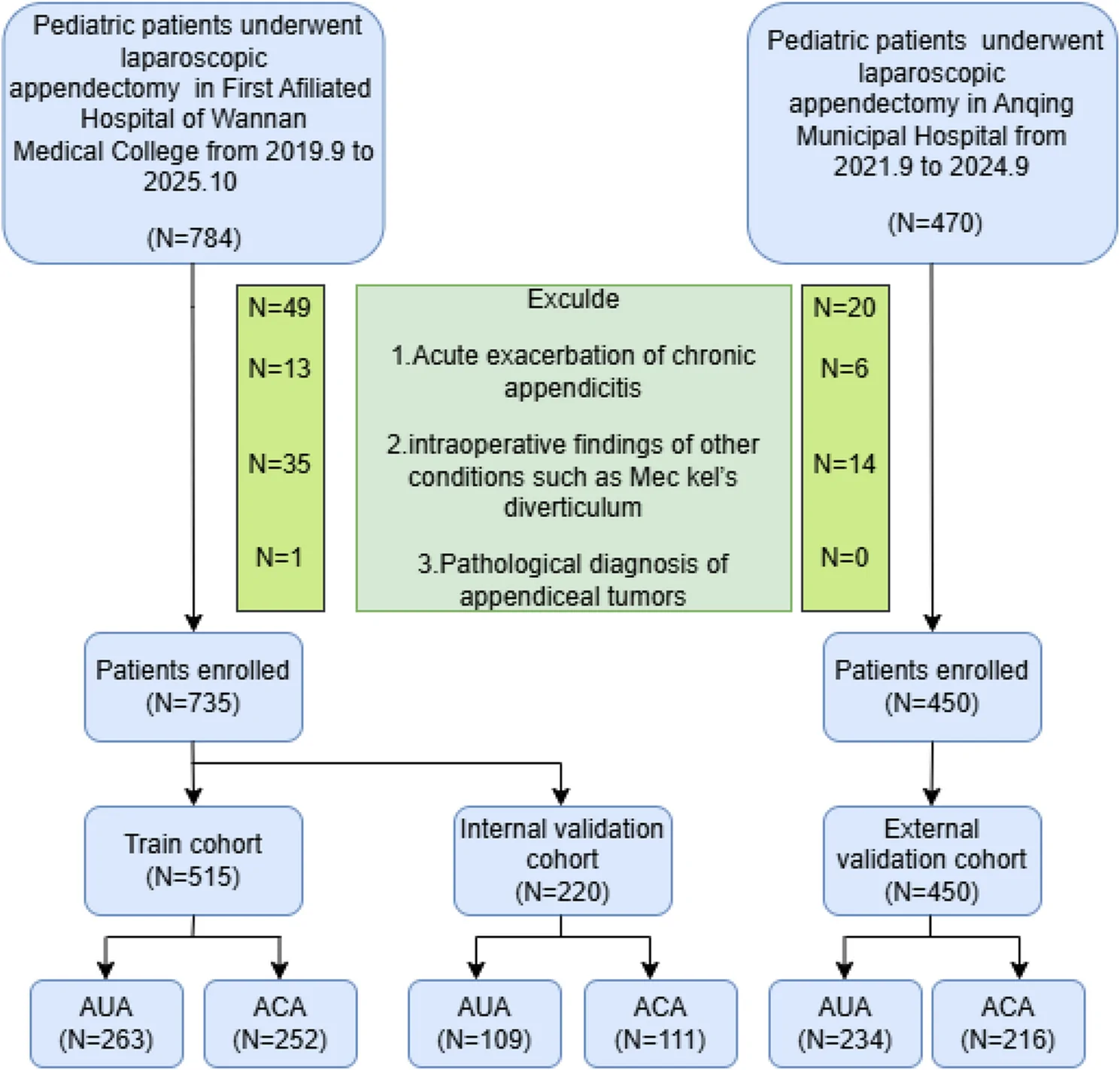
Flowchart for patient selection

The study flowchart is presented in Fig. 1. The baseline demographic and clinical characteristics of the study population for the training cohort (*N* = 515), the internal validation cohort (*N* = 220), and the external validation cohort (*N* = 450) are summarized in Table 1. The distribution of most variables was comparable across the cohorts, indicating successful randomization and similar patient populations. Leukocyte, neutrophil, and monocyte counts, as well as lipase levels, exhibited significant variation (*p* < 0.001). However, none were identified as independent predictive factors. Univariate analyses were performed to present the baseline characteristics of patients stratified by appendicitis severity across the threecohorts. These analyses consistently identified a distinct profile associated withACA, characterized by more pronounced systemic inflammatory responses, specific imaging findings, and electrolyte disturbances in all three independent patient cohorts (Supplementary Table 1).

**Table 1:** Patient demographics and baseline characteristics

Characteristic	TC *N* = 515^1^	IVC *N* = 220^1^	EVC *N* = 450^1^	*p*-value^2^
Gender				0.202
Female	177 (34.4%)	82 (37.3%)	138 (30.7%)	
Male	338 (65.6%)	138 (62.7%)	312 (69.3%)	
Age	8.60 (6.40, 11.10)	8.80 (6.70, 11.30)	8.60 (6.70, 10.50)	0.480
BMI	18.00 (16.00, 19.00)	18.00 (16.00, 19.00)	18.00 (17.00, 19.00)	0.534
Onset time	24 (12, 48)	24 (19, 48)	26 (18, 42)	0.117
Fever(≥38℃）				0.698
no	274 (53.2%)	110 (50.0%)	239 (53.1%)	
yes	241 (46.8%)	110 (50.0%)	211 (46.9%)	
Emesis				0.527
no	199 (38.6%)	81 (36.8%)	185 (41.1%)	
yes	316 (61.4%)	139 (63.2%)	265 (58.9%)	
Peritoneal irritation signs				0.691
no	52 (10.1%)	23 (10.5%)	53 (11.8%)	
yes	463 (89.9%)	197 (89.5%)	397 (88.2%)	
Leukocyte count	16 (12, 19)	15 (12, 19)	18 (12, 26)	<0.001
Neutrophil count	14 (10, 17)	13 (10, 17)	16 (10, 24)	<0.001
Lymphocyte count	1.20 (0.80, 1.80)	1.20 (0.80, 1.70)	1.30 (0.90, 1.70)	0.258
NLR	11 (7, 18)	11 (7, 17)	14 (9, 19)	0.002
monocyte count	0.70 (0.50, 1.00)	0.70 (0.50, 0.90)	0.80 (0.60, 1.00)	<0.001
Hemoglobin	133 (126, 140)	131 (125, 139)	133 (125, 144)	0.051
Platelet count	274 (231, 324)	264 (225, 322)	282 (230, 332)	0.311
CRP	38 (10, 95)	49 (11, 86)	30 (15, 92)	0.614
Serum potassium	4.00 (3.79, 4.22)	3.94 (3.69, 4.19)	3.90 (3.70, 4.20)	0.013
Serum sodium	136.4 (134.0, 138.8)	136.7 (134.0, 138.9)	136.6 (134.2, 138.8)	0.447
Serum chloride	100.3 (97.9, 102.6)	100.2 (97.8, 102.8)	100.1 (97.3, 102.4)	0.551
Fibrinogen	4.16 (3.24, 5.38)	4.11 (3.29, 4.96)	4.59 (3.11, 5.85)	0.148
Lactic dehydrogenase	246 (213, 273)	242 (210, 266)	251 (216, 281)	0.029
Lipase	20 (16, 28)	20 (16, 27)	26 (18, 33)	<0.001
Carbon dioxide combining power	20.00 (18.00, 21.80)	20.30 (18.10, 21.85)	20.00 (18.00, 21.70)	0.944
Blood types				0.912
A	166 (32.2%)	66 (30.0%)	137 (30.4%)	
B	142 (27.6%)	58 (26.4%)	126 (28.0%)	
O	159 (30.9%)	78 (35.5%)	142 (31.6%)	
AB	48 (9.3%)	18 (8.2%)	45 (10.0%)	
maximal appendix diameter	11.80 (10.80, 12.70)	11.80 (10.90, 12.80)	12.00 (10.90, 13.00)	0.582
Appendicolith				0.379
no	203 (39.4%)	97 (44.1%)	174 (38.7%)	
yes	312 (60.6%)	123 (55.9%)	276 (61.3%)	
Free peritoneal effusion				0.915
no	198 (38.4%)	88 (40.0%)	177 (39.3%)	
yes	317 (61.6%)	132 (60.0%)	273 (60.7%)	
Periappendiceal fat blurring				0.865
no	199 (38.6%)	89 (40.5%)	180 (40.0%)	
yes	316 (61.4%)	131 (59.5%)	270 (60.0%)	
Local abscess				0.630
no	496 (96.3%)	214 (97.3%)	431 (95.8%)	
yes	19 (3.7%)	6 (2.7%)	19 (4.2%)	
Ileus				0.582
no	485 (94.2%)	205 (93.2%)	428 (95.1%)	
yes	30 (5.8%)	15 (6.8%)	22 (4.9%)	
Enlarged lymph nodes around the ileocecal region				0.984
no	252 (48.9%)	107 (48.6%)	222 (49.3%)	
yes	263 (51.1%)	113 (51.4%)	228 (50.7%)	

*TC* Training Cohort, *IVC *Internal Validation Cohort, *EVC* External Validation Cohort

^1^ *n* (%); Median (Q1, Q3)

^2^ Pearson's Chi-squared test; Kruskal-Wallis rank sum test

### Predictive model

**Fig. 2:**
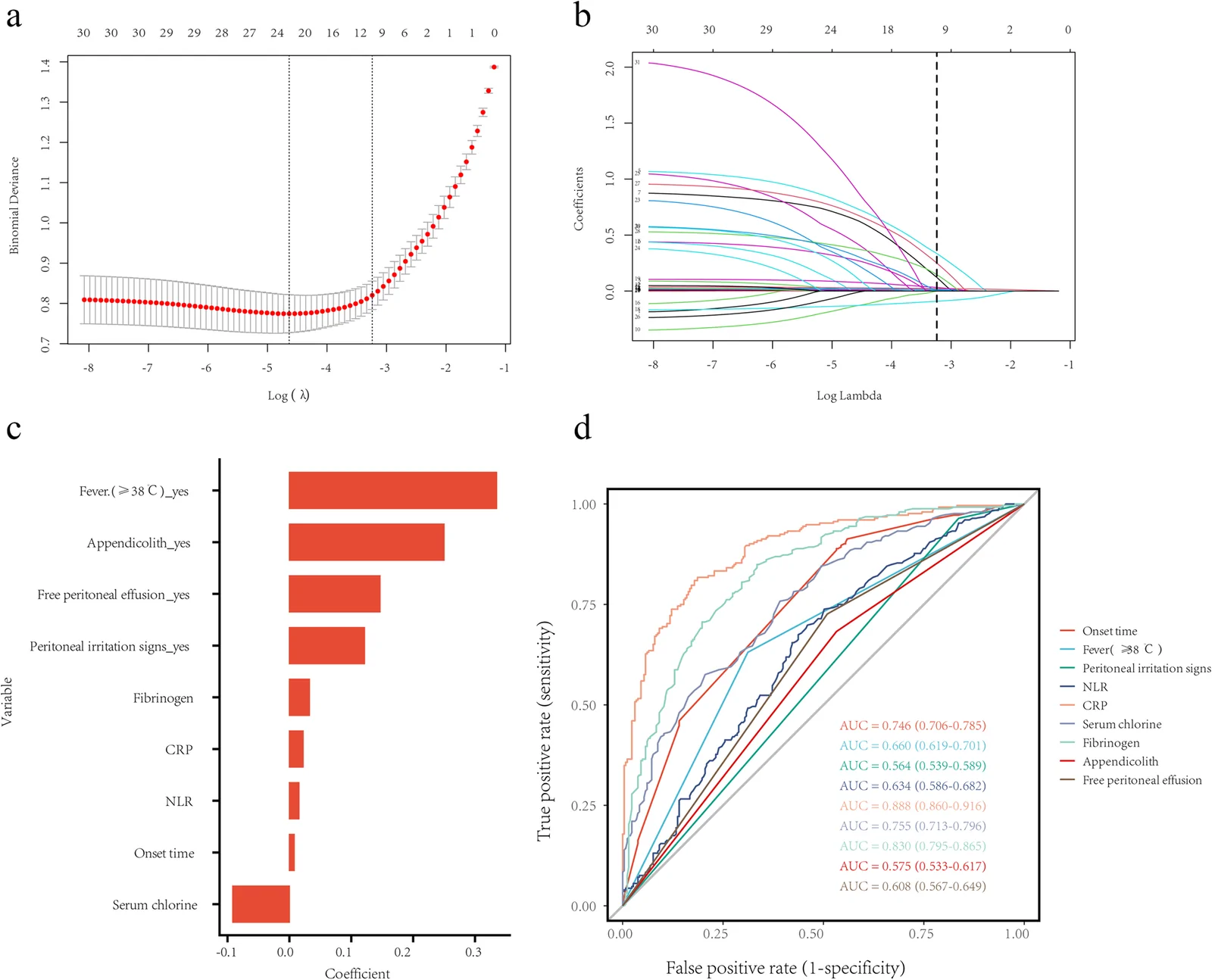
**a** 10-fold cross-validation was applied to select the most suitable feature using the Lasso regression model. (λ = 0.0391537232095145). **b** Plot of the Lasso regression coefficient profiles (λ = 0.0391537232095145). **c** Lasso-Selected Predictors and CorrespondingCoefficients. Only variables with non-zero coefficients after Lasso selection are shown. **d** Comparison of ROC curves for Individual Predictive Variables. ROC curves for individual predictors assessed in univariate logistic regression

In the training cohort, LASSO regression analysis was employed to select the most valuable predictors from the initial set of 29 candidate variables. A 10-fold cross-validation identified an optimal λ value of 0.039, resulting in nine predictors with non-zero coefficients (Fig. 2a and b; Supplementary Table 2). These nine features were: onset time, fever (≥ 38 °C), peritoneal irritation signs, NLR, CRP, serum chloride, fibrinogen, appendicolith, and free peritoneal effusion (Fig. 2c). Univariate analysis confirmed that each selected predictor had a statistically significant—though varied—ability to discriminate between ACA and AUA, as demonstrated by their individual ROC curves (Fig. 2d).

### Multivariate logistic analyses

**Fig. 3:**
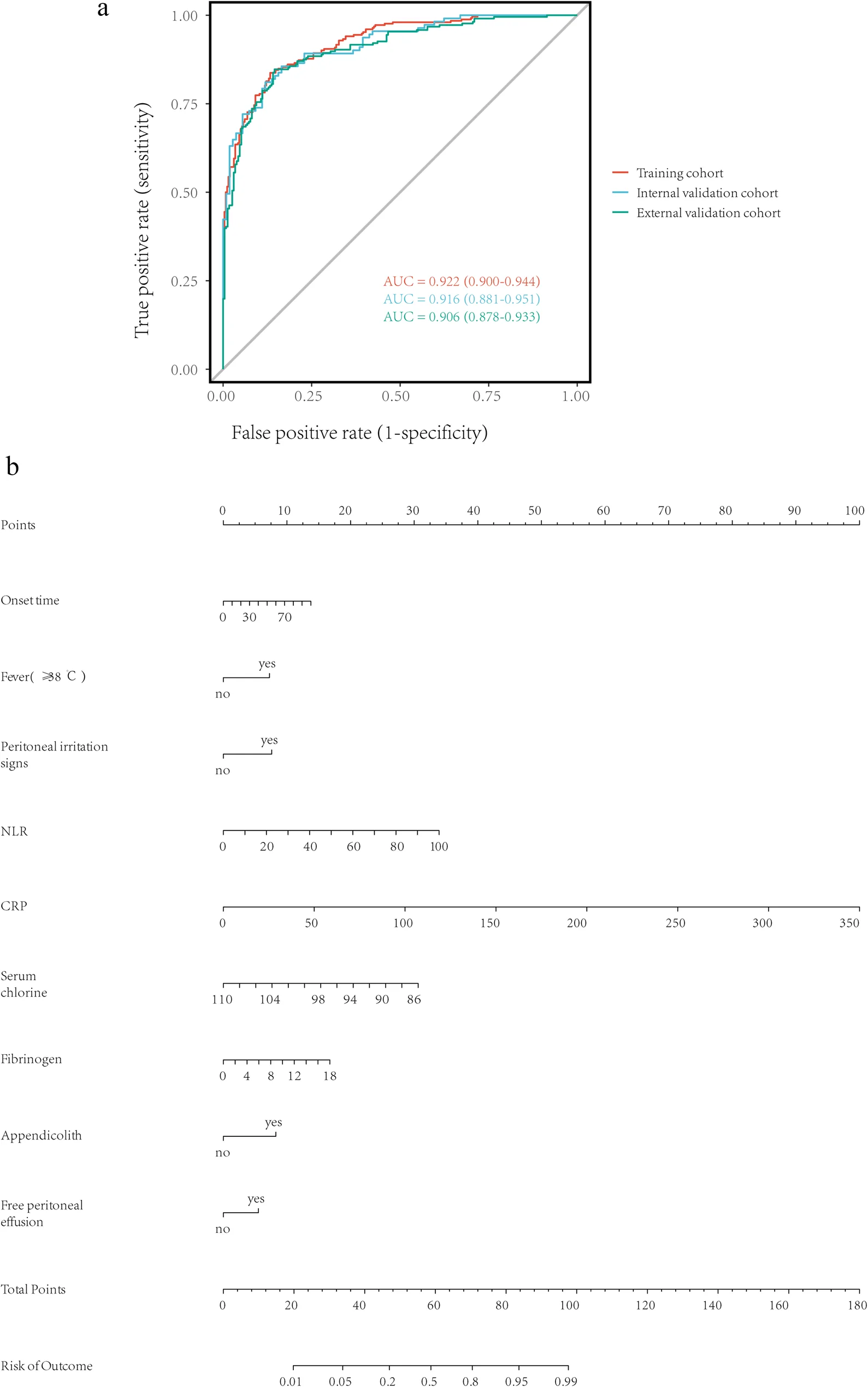
**a** AUCs of the model in the three cohorts. **b** Nomogram prediction model. Onset time(hours): 0-100; Fever(℃): ≥38; NLR(%): 0-100; CRP(mg/L): 0-350; Serum cholerine(mmmol/L): 110 − 86; Fibrinogen(g/L); 0–18

These nine selected predictors were incorporated into a multivariate logistic regression model in the training cohort (Table 2). The analysis confirmed that fever (≥ 38 °C), NLR, CRP, serum chloride level, appendicolith, and free peritoneal effusion were significant independent predictors of ACA (*p* < 0.05). Variables selected by the LASSO algorithm were retained in the final multivariable modelregardless of their individual p-values, as LASSO identifies the optimal combination of predictors that maximizes overall model performance and generalizability, rather than relying on traditional significance testing. Therefore, onset time peritoneal irritation signs, and fibrinogen level did not reach statistical significance in the multivariate model, they were also retained based on their selection by the LASSO algorithm and their established clinical relevance, thereby contributing to a comprehensive predictive model. Based on this final model, a predictive nomogram was constructed to provide a quantitative and visual tool for clinical use (Fig. 3b), which is also available online at https://HongLongMa.shinyapps.io/dynnomapp.

**Table 2:** Results of multivariate logistic regression for the training cohort

Characteristic	*N*	Event N	OR	95% CI	*p*-value
Onset time	515	252	1.02	1.00, 1.03	0.056
Fever(≥38℃）					
no	274	93	—	—	
yes	241	159	2.35	1.38, 4.04	0.002
Peritoneal irritation signs					
no	52	9	—	—	
yes	463	243	2.45	0.94, 7.17	0.082
NLR	515	252	1.04	1.02, 1.07	0.002
CRP	515	252	1.03	1.03, 1.04	<0.001
Serum chloride	515	252	0.86	0.78, 0.94	0.002
Fibrinogen	515	252	1.12	0.92, 1.34	0.228
Appendicolith					
no	203	80	—	—	
yes	312	172	2.65	1.52, 4.72	<0.001
Free peritoneal effusion					
no	198	69	—	—	
yes	317	183	1.92	1.11, 3.33	0.020

### Nomogram and ROC curve analysis

The predictive performance of the nomogram was evaluated in all three cohortsusing ROC curve analysis (Fig. 3a). To use the nomogram, a vertical line is drawn from the value of each predictor to the ‘Points’ axis to determine the individual score. The scores for all nine predictors are then summed to obtain the’Total Points’. Finally, a vertical line is drawn from the ‘Total Points’ axis to the ‘Risk of ACA’ axis to estimate the individual patient’s probability of having ACA. These analyses demonstrated the model’s robust predictive ability across all cohorts. The training cohort achieved an area under the curve (AUC) of 0.922 (95% CI: 0.900–0.944), the internal validation cohort exhibited an AUC of0.916 (95% CI: 0.881–0.951), and the external validation cohort yielded an AUC of 0.906 (95% CI: 0.878–0.933). These results indicate strong and consistentdiscriminative performance. The model’s classification accuracy was also assessed at multiple risk cutoffs across three cohorts (Supplementary Tables 3, 4 and 5).

### The calibration curve and DCA

**Fig. 4:**
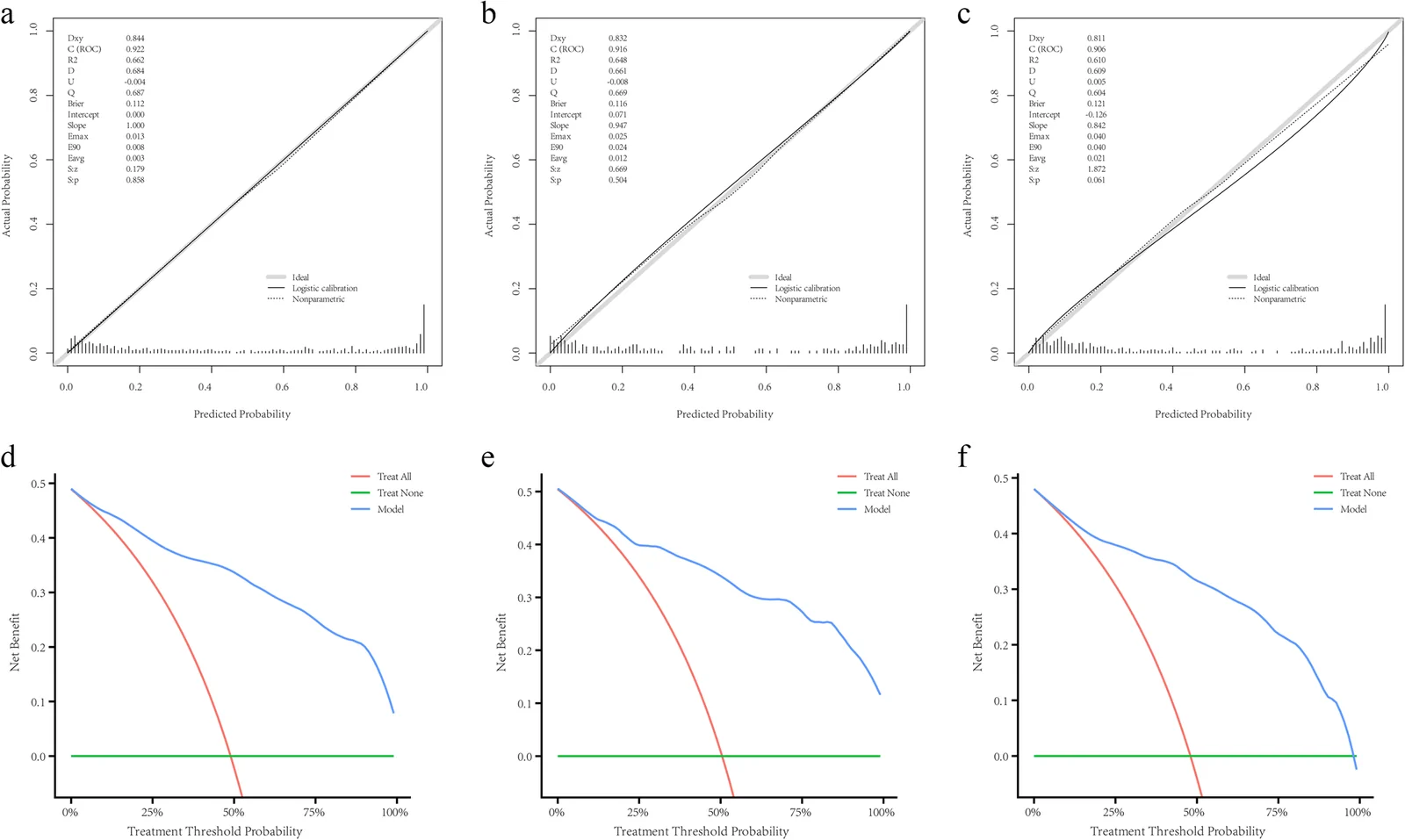
**a** Calibration curve of the nomogram prediction model for the training cohort, (**b**) calibration curve of the nomogram prediction model for the internal validation cohort, (**c**) calibration curve of the nomogram prediction model for the external validation cohort, (**d**) decision curve analysis of the nomogram prediction model for the training cohort, (**e**) decision curve analysis of the nomogram prediction model for the internal validation cohort, (**f**) decision curve analysis of the nomogram prediction model for the external validation cohort

Calibration curves for the nomogram showed strong agreement between predicted probabilities and actual outcomes in all three cohorts (Fig. 4a–c), with non-significant p‑values from the Hosmer–Lemeshow test (*p* > 0.05), indicating that the model is well calibrated. Furthermore, DCA was performed to assess the clinical utility of the nomogram (Fig. 4d–f). The results demonstrated that using the nomogram to guide clinical decisions provides a substantial net benefit acrossa wide range of threshold probabilities compared to the strategies of treating all or no patients, supporting its value in clinical practice. (Tables and figures are placed at the end of the manuscript.)

## Discussion

In this multicenter study, we developed and externally validated a nomogram that accurately predicts the risk of ACA in children using nine readily availablepreoperative variables. The model demonstrated excellent discrimination (AUC > 0.90) and calibration in both internal and external validation cohorts, highlighting its potential as a robust clinical decision-support tool.

Our model’s performance compares favorably with existing tools. Traditional scoring systems such as the PAS and Alvarado score, while useful for diagnosis, lack the specificity required for reliable severity stratification [21, 22]. Recently, more advanced models have emerged. For example, the SAS 2.0 score, developed by Scheijmans et al., showed high accuracy but was validated primarily inan adult population [23]. The PRE_APP study by van Amstel et al. developed apediatric-specific model but did not include an external validation cohort, limiting its generalizability [24]. Our study contributes to the literature by presenting a model developed using a machine-learning approach (LASSO) for objective feature selection. The primary advantage of LASSO lies in its superior ability to handle multicollinearity and prevent overfitting, resulting in a more robust and generalizable model compared to stepwise selection [25]. While a single CART model is visually intuitive, LASSO provides a more stable and often more practically interpretable output for clinical use, especially when the underlying relationships are approximately linear [26]. A review by Rudin strongly argues against using black-box models for high-stakes decisions, emphasizing that interpretable models such as LASSO can often achieve comparable performance whileproviding the necessary transparency for accountability and trust [27]. Furthermore, the simplicity of a linear model like LASSO facilitates easier validation, calibration, and integration into existing clinical workflows, such as electronic health record systems, compared to more computationally complex ensemble methods [28]. Most importantly, we demonstrate its robustness through rigorous external validation in a large, independent pediatric cohort. This addresses a critical gap in the translation of predictive models into widespread clinical practice.

The nine predictors identified by our model—including inflammatory markers (NLR, CRP), metabolic indicators (serum chloride), coagulation factors (fibrinogen), clinical signs (fever ≥ 38 °C, onset time, peritoneal irritation signs), and imaging findings (appendicolith, free peritoneal effusion)—are pathophysiologically plausible. Notably, our finding that decreased serum chloride is an independent predictor of ACA aligns with emerging evidence linking hypochloremia to severe inflammation and sepsis, potentially through its role in inflammasome activation [19, 29]. Chloride ion channels have been implicated in the activation of inflammatory pathways, including the NLRP3 inflammasome [30]. Chloride ions also play a regulatory role in the activation and release of interleukin-1β (IL-1β) [31]. Inflammatory conditions can activate volume-sensitive chloride channels, and subsequent IL-1β release further promotes channel opening, contributing to mitochondrial dysfunction and lysosomal damage—key mechanisms in NLRP3 inflammasome activation [32]. The observed association between reduced chloridelevels and inflammatory intensity is consistent with the low serum chloride levels identified in patients with CA in our study. This underscores the value of incorporating a broad range of biomarkers in the initial assessment.

The primary clinical utility of this nomogram is to facilitate preoperative risk stratification. A high predicted probability of ACA substantially influences treatment strategy and prognosis in pediatric acute appendicitis. For example, a high-risk score could prompt surgeons to prioritize urgent operative intervention, potentially mitigating the reported 1–2% hourly increase in perforation risk associated with in-hospital delay [9]. It also enables more detailed preoperative counseling with families regarding the likelihood of a complicated postoperative course.The user-friendly web-based nomogram (https://HongLongMa.shinyapps.io/dynnomapp) allows seamless integration into clinical workflow, offering real-time, individualized risk assessment at the bedside. The web-based application is freely accessible without the need for user registration. It is important to note that while the tool has undergone robust retrospective external validation, it has not yet been subjected to prospective clinical testing. Future prospective trials are required to evaluate its real-time impact on clinical decision-making.

This study has several strengths, including its multicenter design, large sample size, robust external validation, and the use of LASSO regression for objective variable selection. However, several limitations must be acknowledged. First, itsretrospective nature is susceptible to information and selection bias. Our cohort represents a population at a tertiary referral center, which typically manages cases with higher baseline severity or those transferred after inconclusive initial evaluations at primary centers. Therefore, this study population may not fully reflect routine pediatric emergency practice where ultrasound is the primary modality and non-operative management is more prevalent. Second, including only patients who underwent CT scans is a major limitation. While imaging findings on CT—such as appendicolith and free peritoneal fluid—are well-established as significant diagnostic indicators of ACA [33], this protocol likely introduced selection bias toward a cohort with higher baseline severity. These predictors are routinely and accurately detectable by transabdominal ultrasound. Therefore, prospective validation using ultrasound-derived data is required to confirm this. Moreover, this approach is not aligned with current guidelines, which recommend ultrasound as the first-line imaging modality to minimize radiation exposure in children. The model’s performance in a population screened primarily by ultrasound requires prospective validation. Third, the age cutoff of ≤ 14 years, while reflecting local practice, may limit the model’s applicability in regions with different pediatric age definitions. Fourth, this study excluded patients with periappendiceal abscess because they were typically managed non-operatively and lacked pathological confirmation [34]. This exclusion may restrict the nomogram’s generalizability across the full clinical spectrum of acute complicated appendicitis. To address these limitations, future research should employ prospective multicenter cohort studies across diverse healthcare settings to externally validate these findings and enhance their global applicability. An interdisciplinary framework integrating social medicine, health systems research, and data science is also warranted. Finally, incorporating novel biomarkers—such as systemic immune-inflammation indices, ischemia-modified albumin, or microbial profiles—could further improve the model’s predictive accuracy.

## Conclusion

In conclusion, this study presents an externally validated nomogram that effectively predicts complicated appendicitis in children using common clinical parameters. Despite its limitations, the model demonstrates strong performance and has the potential to be a valuable tool for preoperative risk stratification. Future research should focus on prospectively validating this model in broader clinical settings—particularly in cohorts where ultrasound is the primary diagnostic imaging tool—to confirm its clinical utility and impact on patient outcomes.

## Supplementary Information

Supplementary Material 1.

## Data Availability

Research data supporting this publication are available from the corresponding author on reasonable request.

## Abbreviations

ACA: Acute complicated appendicitis
AUA: Acute uncomplicated appendicitis
CRP: C-reactive protein
CT: Computed tomography
BMI: Body mass index
NLR: Neutrophil-lymphocyte ratio
CIs: Confidence intervals
LASSO: Least absolute shrinkage and selection operator
ROC: Receiver operating characteristic
DCA: Decision curve analysis
AUC: The area under the ROC curve
OR: Odds ratio
