# Supplementary material for: Integration of clinical information for identification of gangrenous or perforated appendicitis in children: a multicenter retrospective cohort study with external validation
Source: BMC Pediatr. 2026 Jul 21;26:881. doi: 10.1186/s12887-026-07314-3 (PMC13584546; doi:10.1186/s12887-026-07314-3)
Supplement: Supplementary file 1 [file 12887_2026_7314_MOESM1_ESM.docx]

Supplementary Table 1 Univariate analyses were used to compare the indices between different outcome groups in the following table.

Patient demographics and baseline characteristics

| **Characteristic** | **Cohort (Training Cohort), N = 515** | | | **Cohort (Internal validation Cohort), N = 220** | | | **Cohort (External validation Cohort), N = 450** | | |
| --- | --- | --- | --- | --- | --- | --- | --- | --- | --- |
|  | **AUA  N = 263^1^** | **ACA  N = 252^1^** | **p-value^2^** | **AUA  N = 109^1^** | **ACA  N = 111^1^** | **p-value^3^** | **AUA  N = 234^1^** | **(ACA  N = 216^1^** | **p-value^2^** |
| **Gender** |  |  | 0.415 |  |  | 0.065 |  |  | 0.961 |
| Female | 86 (32.7%) | 91 (36.1%) |  | 34 (31.2%) | 48 (43.2%) |  | 72 (30.8%) | 66 (30.6%) |  |
| Male | 177 (67.3%) | 161 (63.9%) |  | 75 (68.8%) | 63 (56.8%) |  | 162 (69.2%) | 150 (69.4%) |  |
| **Age(years)** | 8.80 (6.60, 11.50) | 8.40 (6.30, 10.60) | 0.031 | 9.50 (7.50, 11.60) | 7.60 (5.80, 10.70) | <0.001 | 8.90 (6.90, 10.80) | 8.60 (6.55, 10.10) | 0.124 |
| **BMI(Kg/m2)** | 18.00 (16.00, 20.00) | 18.00 (16.00, 19.00) | 0.045 | 18.00 (17.00, 19.00) | 17.00 (16.00, 19.00) | 0.004 | 18.00 (17.00, 19.00) | 18.00 (17.00, 19.00) | 0.906 |
| **Onset time(hours)** | 24 (12, 24) | 24 (24, 48) | <0.001 | 24 (12, 24) | 30 (24, 48) | <0.001 | 23 (15, 30) | 38 (24, 51) | <0.001 |
| **Fever(≥38℃）** |  |  | <0.001 |  |  | <0.001 |  |  | <0.001 |
| no | 181 (68.8%) | 93 (36.9%) |  | 70 (64.2%) | 40 (36.0%) |  | 154 (65.8%) | 85 (39.4%) |  |
| yes | 82 (31.2%) | 159 (63.1%) |  | 39 (35.8%) | 71 (64.0%) |  | 80 (34.2%) | 131 (60.6%) |  |
| **Emesis** |  |  | 0.025 |  |  | 0.055 |  |  | 0.014 |
| no | 114 (43.3%) | 85 (33.7%) |  | 47 (43.1%) | 34 (30.6%) |  | 109 (46.6%) | 76 (35.2%) |  |
| yes | 149 (56.7%) | 167 (66.3%) |  | 62 (56.9%) | 77 (69.4%) |  | 125 (53.4%) | 140 (64.8%) |  |
| **Peritoneal irritation signs** |  |  | <0.001 |  |  | <0.001 |  |  | <0.001 |
| no | 43 (16.3%) | 9 (3.6%) |  | 22 (20.2%) | 1 (0.9%) |  | 43 (18.4%) | 10 (4.6%) |  |
| yes | 220 (83.7%) | 243 (96.4%) |  | 87 (79.8%) | 110 (99.1%) |  | 191 (81.6%) | 206 (95.4%) |  |
| **Leukocyte count(×109/L)** | 15.3 (12.0, 18.5) | 16.3 (12.3, 20.5) | 0.020 | 14.8 (11.3, 17.9) | 16.3 (12.7, 19.7) | 0.025 | 19 (12, 27) | 18 (12, 25) | 0.334 |
| **Neutrophil count(×109/L)** | 13.0 (9.5, 16.2) | 14.6 (10.5, 18.4) | 0.003 | 12.9 (9.3, 15.1) | 14.0 (11.0, 18.0) | 0.011 | 16 (10, 24) | 16 (10, 23) | 0.490 |
| **Lymphocyte count(×109/L)** | 1.40 (0.90, 2.10) | 1.10 (0.70, 1.50) | <0.001 | 1.40 (0.90, 1.90) | 1.10 (0.70, 1.50) | 0.002 | 1.60 (1.10, 2.00) | 1.10 (0.80, 1.40) | <0.001 |
| **NLR** | 9 (6, 16) | 13 (8, 21) | <0.001 | 9 (6, 15) | 13 (8, 23) | <0.001 | 13 (9, 16) | 18 (9, 25) | <0.001 |
| **monocyte count(×109/L)** | 0.70 (0.50, 0.90) | 0.70 (0.50, 1.00) | 0.196 | 0.70 (0.50, 0.90) | 0.70 (0.50, 1.00) | 0.340 | 0.80 (0.60, 1.00) | 0.90 (0.60, 1.20) | <0.001 |
| **Hemoglobin(g/L)** | 132 (126, 139) | 134 (126, 141) | 0.491 | 131 (126, 138) | 133 (124, 140) | 0.751 | 135 (124, 146) | 133 (126, 141) | 0.153 |
| **Platelet count(×109/L)** | 272 (228, 322) | 280 (233, 325) | 0.325 | 259 (221, 303) | 265 (228, 341) | 0.176 | 286 (223, 331) | 279 (239, 333) | 0.597 |
| **CRP(mg/L)** | 13 (3, 33) | 90 (49, 137) | <0.001 | 11 (3, 34) | 83 (56, 132) | <0.001 | 18 (10, 28) | 91 (53, 131) | <0.001 |
| **Serum potassium(mmol/L)** | 4.01 (3.79, 4.23) | 4.00 (3.78, 4.22) | 0.532 | 3.94 (3.75, 4.19) | 3.93 (3.66, 4.18) | 0.873 | 4.00 (3.80, 4.20) | 3.90 (3.60, 4.10) | 0.021 |
| **Serum sodium(mmol/L)** | 137.8 (135.5, 139.8) | 135.0 (132.3, 137.3) | <0.001 | 137.3 (135.3, 139.6) | 135.5 (133.0, 138.2) | <0.001 | 137.6 (135.2, 139.7) | 135.7 (133.2, 137.9) | <0.001 |
| **Serum chlorine(mmol/L)** | 101.8 (99.4, 103.7) | 98.8 (96.5, 100.8) | <0.001 | 101.6 (99.4, 103.9) | 99.1 (96.6, 100.8) | <0.001 | 101.4 (98.9, 104.2) | 98.7 (96.2, 100.8) | <0.001 |
| **Fibrinogen(g/L)** | 3.39 (2.76, 4.21) | 5.00 (4.20, 6.72) | <0.001 | 3.36 (2.63, 4.07) | 4.86 (4.16, 6.26) | <0.001 | 3.73 (2.44, 4.95) | 5.47 (4.38, 6.78) | <0.001 |
| **Lactic dehydrogenase(U/L)** | 247 (213, 272) | 246 (212, 274) | 0.580 | 242 (214, 265) | 241 (208, 272) | 0.960 | 252 (216, 281) | 249 (217, 282) | 0.885 |
| **Lipase(U/L)** | 21 (17, 30) | 19 (15, 26) | 0.003 | 23 (17, 31) | 19 (15, 23) | <0.001 | 26 (18, 34) | 25 (17, 32) | 0.169 |
| **Carbon dioxide combining power(mmol/L)** | 20.50 (18.80, 22.00) | 19.45 (17.40, 21.50) | <0.001 | 20.80 (19.10, 22.40) | 19.40 (17.60, 21.40) | <0.001 | 20.2 (18.2, 21.5) | 20.0 (17.6, 21.8) | 0.480 |
| **Blood tpyes** |  |  | 0.424 |  |  | 0.146 |  |  | 0.629 |
| A | 89 (33.8%) | 77 (30.6%) |  | 34 (31.2%) | 32 (28.8%) |  | 73 (31.2%) | 64 (29.6%) |  |
| B | 65 (24.7%) | 77 (30.6%) |  | 24 (22.0%) | 34 (30.6%) |  | 62 (26.5%) | 64 (29.6%) |  |
| O | 86 (32.7%) | 73 (29.0%) |  | 38 (34.9%) | 40 (36.0%) |  | 72 (30.8%) | 70 (32.4%) |  |
| AB | 23 (8.7%) | 25 (9.9%) |  | 13 (11.9%) | 5 (4.5%) |  | 27 (11.5%) | 18 (8.3%) |  |
| **maximal appendix diameter(mm)** | 11.80 (10.90, 12.70) | 11.90 (10.70, 12.80) | 0.749 | 11.80 (11.20, 12.60) | 11.80 (10.70, 13.40) | 0.715 | 12.10 (11.00, 13.00) | 12.00 (10.90, 12.90) | 0.133 |
| **Appendicolith** |  |  | <0.001 |  |  | 0.107 |  |  | 0.001 |
| no | 123 (46.8%) | 80 (31.7%) |  | 54 (49.5%) | 43 (38.7%) |  | 107 (45.7%) | 67 (31.0%) |  |
| yes | 140 (53.2%) | 172 (68.3%) |  | 55 (50.5%) | 68 (61.3%) |  | 127 (54.3%) | 149 (69.0%) |  |
| **Free peritoneal effusion** |  |  | <0.001 |  |  | <0.001 |  |  | <0.001 |
| no | 129 (49.0%) | 69 (27.4%) |  | 56 (51.4%) | 32 (28.8%) |  | 117 (50.0%) | 60 (27.8%) |  |
| yes | 134 (51.0%) | 183 (72.6%) |  | 53 (48.6%) | 79 (71.2%) |  | 117 (50.0%) | 156 (72.2%) |  |
| **Periappendiceal fat blurring** |  |  | <0.001 |  |  | <0.001 |  |  | 0.010 |
| no | 127 (48.3%) | 72 (28.6%) |  | 57 (52.3%) | 32 (28.8%) |  | 107 (45.7%) | 73 (33.8%) |  |
| yes | 136 (51.7%) | 180 (71.4%) |  | 52 (47.7%) | 79 (71.2%) |  | 127 (54.3%) | 143 (66.2%) |  |
| **Local abscess** |  |  | 0.028 |  |  | 0.212 |  |  | 0.001 |
| no | 258 (98.1%) | 238 (94.4%) |  | 108 (99.1%) | 106 (95.5%) |  | 231 (98.7%) | 200 (92.6%) |  |
| yes | 5 (1.9%) | 14 (5.6%) |  | 1 (0.9%) | 5 (4.5%) |  | 3 (1.3%) | 16 (7.4%) |  |
| **Ileus** |  |  | <0.001 |  |  | 0.004 |  |  | <0.001 |
| no | 260 (98.9%) | 225 (89.3%) |  | 107 (98.2%) | 98 (88.3%) |  | 233 (99.6%) | 195 (90.3%) |  |
| yes | 3 (1.1%) | 27 (10.7%) |  | 2 (1.8%) | 13 (11.7%) |  | 1 (0.4%) | 21 (9.7%) |  |
| **Enlarged lymph nodes around the ileocecal region** |  |  | 0.101 |  |  | 0.592 |  |  | 0.002 |
| no | 138 (52.5%) | 114 (45.2%) |  | 55 (50.5%) | 52 (46.8%) |  | 132 (56.4%) | 90 (41.7%) |  |
| yes | 125 (47.5%) | 138 (54.8%) |  | 54 (49.5%) | 59 (53.2%) |  | 102 (43.6%) | 126 (58.3%) |  |
| BMI, body mass index; NLR, neutrophil-lymphocyte ratio; CRP, C-reactive protein; AUA, acute uncomplicated appendicitis; ACA, acute complicated appendicitis.  ^1^n (%); Median (Q1, Q3) | | | | | | | | | |
| ^2^Pearson's Chi-squared test; Wilcoxon rank sum test | | | | | | | | | |
| ^3^Pearson's Chi-squared test; Wilcoxon rank sum test; Fisher's exact test | | | | | | | | | |

Supplementary table 2 nine preoperative features with non-zero coefficients were selected as predictive factors for pediatric ACA

The coefficients of Lasso regression analysis

| variable | Coefficient |
| --- | --- |
| (Intercept) | 6.866830335 |
| Gender_Male | 0.000000000 |
| Age.years. | 0.000000000 |
| BMI.Kg.m2. | 0.000000000 |
| Onset.time.hours. | 0.007697461 |
| Fever..38.._yes | 0.334951907 |
| Emesis_yes | 0.000000000 |
| Peritoneal.irritation.signs_yes | 0.121393548 |
| Leukocyte.count..109.L. | 0.000000000 |
| Neutrophil.count..109.L. | 0.000000000 |
| Lymphocyte.count..109.L. | 0.000000000 |
| NLR | 0.015448128 |
| molocyte.count..109.L. | 0.000000000 |
| Hemoglobin.g.L. | 0.000000000 |
| Platelet.count..109.L. | 0.000000000 |
| CRP.mg.L. | 0.022199517 |
| Serum.potassium.mmol.L. | 0.000000000 |
| Serum.sodium.mmol.L. | 0.000000000 |
| Serum.chlorine.mmol.L. | -0.091607173 |
| Fibrinogen.g.L. | 0.032323812 |
| Lactic.dehydrogenase.U.L. | 0.000000000 |
| Lipase.U.L. | 0.000000000 |
| Carbon.dioxide.combining.power.mmol.L. | 0.000000000 |
| Blood.tpyes_B | 0.000000000 |
| Blood.tpyes_O | 0.000000000 |
| Blood.tpyes_AB | 0.000000000 |
| maximal.appendix.diameter.mm. | 0.000000000 |
| Appendicolith_yes | 0.249987271 |
| Free.peritoneal.effusion_yes | 0.146545176 |
| Periappendiceal.fat.blurring_yes | 0.000000000 |
| Local.abscess_yes | 0.000000000 |
| Ileus_yes | 0.000000000 |
| Enlarged.lymph.nodes.around.the.ileocecal.region_yes | 0.000000000 |

BMI, body mass index; NLR, neutrophil-lymphocyte ratio; CRP, C-reactive protein

Supplementary table 3 Classification accuracy for prediction at different risk cutoff points for the model in training cohor

Classification accuracy for prediction at different risk cutoff points for the model in training cohor

| Risk score threshold | Linear Predictor Cutoff Point | Sensitivity (%) | Specificity (%) | PPV (%) | NPV (%) | Accuracy (%) | Precision (%) | Recall (%) | F1 |
| --- | --- | --- | --- | --- | --- | --- | --- | --- | --- |
| ≥ 0% | -Inf | 100.0 | 0.0 | 48.9 |  | 48.9 | 48.9 | 100.0 | 0.657 |
| ≥ 10% | -2.1972246 | 98.0 | 44.5 | 62.8 | 95.9 | 70.7 | 62.8 | 98.0 | 0.766 |
| ≥ 20% | -1.3862944 | 94.4 | 63.1 | 71.0 | 92.2 | 78.4 | 71.0 | 94.4 | 0.811 |
| ≥ 30% | -0.8472979 | 89.3 | 73.0 | 76.0 | 87.7 | 81.0 | 76.0 | 89.3 | 0.821 |
| ≥ 40% | -0.4054651 | 85.7 | 81.7 | 81.8 | 85.7 | 83.7 | 81.8 | 85.7 | 0.837 |
| ≥ 50% | 0.0000000 | 82.5 | 86.7 | 85.6 | 83.8 | 84.7 | 85.6 | 82.5 | 0.840 |
| ≥ 60% | 0.4054651 | 77.4 | 90.1 | 88.2 | 80.6 | 83.9 | 88.2 | 77.4 | 0.825 |
| ≥ 70% | 0.8472979 | 69.4 | 94.3 | 92.1 | 76.3 | 82.1 | 92.1 | 69.4 | 0.792 |
| ≥ 80% | 1.3862944 | 59.1 | 97.0 | 94.9 | 71.2 | 78.4 | 94.9 | 59.1 | 0.729 |
| ≥ 90% | 2.1972246 | 51.6 | 98.9 | 97.7 | 68.1 | 75.7 | 97.7 | 51.6 | 0.675 |
| ≥ 100% | Inf | 0.0 | 100.0 |  | 51.1 | 51.1 |  | 0.0 |  |

Supplementary table 4 Classification accuracy for prediction at different risk cutoff points for the model in internal test cohort

Classification accuracy for prediction at different risk cutoff points for the model in internal test cohort

| Risk score threshold | Linear Predictor Cutoff Point | Sensitivity (%) | Specificity (%) | PPV (%) | NPV (%) | Accuracy (%) | Precision (%) | Recall (%) | F1 |
| --- | --- | --- | --- | --- | --- | --- | --- | --- | --- |
| ≥ 0% | -Inf | 100.0 | 0.0 | 50.5 |  | 50.5 | 50.5 | 100.0 | 0.671 |
| ≥ 10% | -2.1972246 | 96.4 | 45.0 | 64.1 | 92.5 | 70.9 | 64.1 | 96.4 | 0.770 |
| ≥ 20% | -1.3862944 | 93.7 | 60.6 | 70.7 | 90.4 | 77.3 | 70.7 | 93.7 | 0.806 |
| ≥ 30% | -0.8472979 | 89.2 | 76.1 | 79.2 | 87.4 | 82.7 | 79.2 | 89.2 | 0.839 |
| ≥ 40% | -0.4054651 | 85.6 | 83.5 | 84.1 | 85.0 | 84.5 | 84.1 | 85.6 | 0.848 |
| ≥ 50% | 0.0000000 | 79.3 | 88.1 | 87.1 | 80.7 | 83.6 | 87.1 | 79.3 | 0.830 |
| ≥ 60% | 0.4054651 | 73.9 | 90.8 | 89.1 | 77.3 | 82.3 | 89.1 | 73.9 | 0.808 |
| ≥ 70% | 0.8472979 | 72.1 | 94.5 | 93.0 | 76.9 | 83.2 | 93.0 | 72.1 | 0.812 |
| ≥ 80% | 1.3862944 | 65.8 | 96.3 | 94.8 | 73.4 | 80.9 | 94.8 | 65.8 | 0.777 |
| ≥ 90% | 2.1972246 | 50.5 | 98.2 | 96.6 | 66.0 | 74.1 | 96.6 | 50.5 | 0.663 |
| ≥ 100% | Inf | 0.0 | 100.0 |  | 49.5 | 49.5 |  | 0.0 |  |

Supplementary table 5 Classification accuracy for prediction at different risk cutoff points for the model in external test cohor

Classification accuracy for prediction at different risk cutoff points for the model in external test cohort

| Risk score threshold | Linear Predictor Cutoff Point | Sensitivity (%) | Specificity (%) | PPV (%) | NPV (%) | Accuracy (%) | Precision (%) | Recall (%) | F1 |
| --- | --- | --- | --- | --- | --- | --- | --- | --- | --- |
| ≥ 0% | -Inf | 100.0 | 0.0 | 50.5 |  | 50.5 | 50.5 | 100.0 | 0.671 |
| ≥ 10% | -2.1972246 | 96.4 | 45.0 | 64.1 | 92.5 | 70.9 | 64.1 | 96.4 | 0.770 |
| ≥ 20% | -1.3862944 | 93.7 | 60.6 | 70.7 | 90.4 | 77.3 | 70.7 | 93.7 | 0.806 |
| ≥ 30% | -0.8472979 | 89.2 | 76.1 | 79.2 | 87.4 | 82.7 | 79.2 | 89.2 | 0.839 |
| ≥ 40% | -0.4054651 | 85.6 | 83.5 | 84.1 | 85.0 | 84.5 | 84.1 | 85.6 | 0.848 |
| ≥ 50% | 0.0000000 | 79.3 | 88.1 | 87.1 | 80.7 | 83.6 | 87.1 | 79.3 | 0.830 |
| ≥ 60% | 0.4054651 | 73.9 | 90.8 | 89.1 | 77.3 | 82.3 | 89.1 | 73.9 | 0.808 |
| ≥ 70% | 0.8472979 | 72.1 | 94.5 | 93.0 | 76.9 | 83.2 | 93.0 | 72.1 | 0.812 |
| ≥ 80% | 1.3862944 | 65.8 | 96.3 | 94.8 | 73.4 | 80.9 | 94.8 | 65.8 | 0.777 |
| ≥ 90% | 2.1972246 | 50.5 | 98.2 | 96.6 | 66.0 | 74.1 | 96.6 | 50.5 | 0.663 |
| ≥ 100% | Inf | 0.0 | 100.0 |  | 49.5 | 49.5 |  | 0.0 |  |
